# RanBPM (RanBP9) regulates mouse c-Kit receptor level and is essential for normal development of bone marrow progenitor cells

**DOI:** 10.18632/oncotarget.13198

**Published:** 2016-11-08

**Authors:** Sandrine Puverel, Erkan Kiris, Satyendra Singh, Kimberly D. Klarmann, Vincenzo Coppola, Jonathan R. Keller, Lino Tessarollo

**Affiliations:** ^1^ Mouse Cancer Genetics Program, Center for Cancer Research, NCI, Frederick, MD 21702, USA; ^2^ Basic Science Program, Leidos Biomedical Research Inc., Frederick National Laboratory for Cancer Research, NCI, Frederick, MD 21702, USA; ^3^ The Ohio State University, Department of Cancer, Biology and Genetics, Wexner Medical Center and James Comprehensive Cancer Center, Columbus, OH 43210, USA

**Keywords:** RanBP9, c-Kit signaling, hematopoietic system, spermatogenesis, stem cells

## Abstract

c-Kit is a tyrosine kinase receptor important for gametogenesis, hematopoiesis, melanogenesis and mast cell biology. Dysregulation of c-Kit function is oncogenic and its expression in the stem cell niche of a number of tissues has underlined its relevance for regenerative medicine and hematopoietic stem cell biology. Yet, very little is known about the mechanisms that control c-Kit protein levels. Here we show that the RanBPM/RanBP9 scaffold protein binds to c-Kit and is necessary for normal c-Kit protein expression in the mouse testis and subset lineages of the hematopoietic system. RanBPM deletion causes a reduction in c-Kit protein but not its mRNA suggesting a posttranslational mechanism. This regulation is specific to the c-Kit receptor since RanBPM reduction does not affect other membrane proteins examined. Importantly, in both mouse hematopoietic system and testis, RanBPM deficiency causes defects consistent with c-Kit loss of expression suggesting that RanBPM is an important regulator of c-Kit function. The finding that this regulatory mechanism is also present in human cells expressing endogenous RanBPM and c-Kit suggests a potential new strategy to target oncogenic c-Kit in malignancies.

## INTRODUCTION

c-Kit is a tyrosine kinase receptor critical for normal mammalian development. Mice with spontaneous mutations at either the *c-kit* locus or the locus of its ligand, stem cell factor (SCF), display very similar and pleiotropic phenotypes, including severe macrocytic anemia, mast cell (MC) deficiency, sterility and pigmentation defects [1]. In addition, c-Kit expression dysregulation plays a central pathogenetic role in the initiation of gastrointestinal stromal tumors, in subsets of melanomas and breast tumors, and in acute myeloid leukemia [reviewed in [2]]. Importantly, c-Kit is one of the critical tyrosine kinase receptors modulating the steady state and long-term maintenance of hematopoietic stem cell progenitors in the adult [3, 4]. The relevance of understanding the mechanisms regulating *c-kit* expression has been further underscored by the recent observation that *c-kit* expression is critical for hematopoietic stem cell (HSC) function in the mature organism and its precise protein level hierarchically organizes different types of HSCs. Thus, HSCs with low levels of surface c-Kit expression and signaling exhibit enhanced self-renewal and long-term reconstitution potential compared with HSCs with high levels of c-Kit [5]. Although the variation in c-Kit expression levels in HSCs reported in this study appears to be regulated by the E3 ubiquitin ligase c-Cbl, it is likely that other unknown mechanisms control c-Kit expression [3]. RanBPM (also called RanBP9) is a scaffold protein that regulates diverse cellular functions through interactions with a wide range of proteins. It has been implicated in a variety of cellular processes including transcription, the regulation of cell morphology, cell adhesion, and the regulation of receptor signaling pathways (reviewed in [6–8]). RanBPM is also involved in pathogenetic events since it affects the processing of the amyloid precursor protein and amyloid β generation [9, 10]. Recently, the generation of mice lacking RanBPM has shown that this gene is not crucial for embryonic development. However, most *RanBPM*^−/−^ mice die post-natally, but surviving mutants have a normal life span although they are significantly smaller than controls [8, 11]. Moreover, *RanBPM*^−/−^ mice are sterile and both males and females display a stage-specific arrest in gametogenesis during the first meiotic division [11]. Interestingly, a point mutation in the *c-kit* gene that specifically abolishes the activation of the PI3-kinase pathway does not affect the number of primordial germ cells (PGC) during embryonic development but leads to defects in spermatogonia proliferation during spermatogenesis at pre-meiotic stages as observed in *RanBPM* deficient males [11–13]. The striking similarity between the phenotypes of *c-kit* and *RanBPM* deficient males prompted us to investigate a possible connection between these genes. We found that RanBPM deletion in testis leads to loss of c-Kit expression and a decrease in PI3-kinase signaling. Moreover, silencing RanBPM expression in erythroid myeloid lymphoid (EML) cells, that require SCF for survival, triggers a decrease in c-Kit expression followed by cell death. Because the SCF/c-Kit system also plays a crucial role in hematopoiesis, we analyzed the bone marrow of *RanBPM*^−/−^ mice and found a significant reduction in all hematopoietic progenitor cell populations that express c-Kit. Altogether, our results demonstrate that RanBPM is a new regulator of c-Kit signaling.

## RESULTS

### c-Kit protein interacts with RanBPM

**Figure 1:**
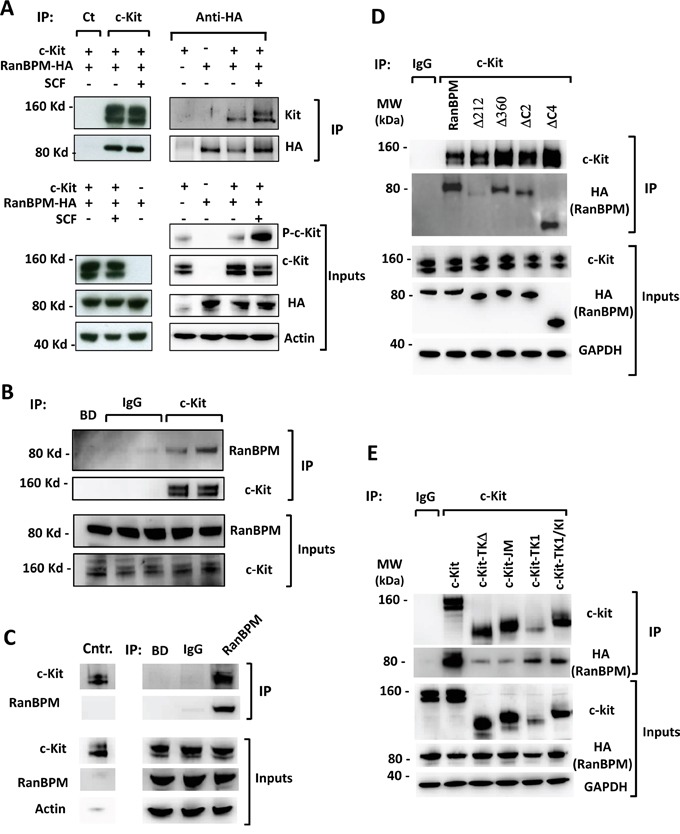
RanBPM interacts with c-Kit through its SPRY domain RanBPM associates with c-Kit in vitro (A) and in vivo (B, C). **A.** protein lysates from HEK293 cells transfected with RanBPM-HA and c-Kit cDNAs and stimulated or not with 100 ng/mL SCF for 30' were subjected to immunoprecipitation (IP) with the indicated antibodies and analyzed by Western blot. A negative control IP was performed using a goat serum isotype (IP: Ct). **B, C.** c-Kit IP RanBPM (B) and RanBPM IP c-kit (C) in mouse testis. Testis lysates from 3 month-old wild type mice were immunoprecipitated with two different c-kit antibodies (B) or an anti-RanBPM antibody (C), control IgG serum isotypes or only beads as negative controls and blotted with the indicated antibodies. **D.** RanBPM interacts with c-Kit through its SPRY domain. HEK293 cells were transfected with vectors expressing a full-length HA-tagged control or c-DNA RANBPM deletion mutants lacking specific RanBPM domains as indicated in Supplementary Figure S2 [48] and subjected to IP experiments with an c-kit antibody and Western analysis with the indicated antibodies. **E.** RanBPM interacts with the tyrosine kinase domain of c-kit. HEK293 cells transfected with vectors expressing a full-length or c-kit c-DNA mutants lacking specific c-Kit intracellular domains as indicated in Supplementary Figure S2 were subjected to IP experiments with an anti-c-Kit antibody and Western analysis with the indicated antibodies. Inputs represent 10% of the lysates used for the IP experiments.

The sterility phenotype observed in the testis of *RanBPM*^−/−^ mice closely resembles that reported in mice with mutations in the c-Kit tyrosine kinase receptor, suggesting a possible link between these two proteins. Moreover, RanBPM interactions with a number of other tyrosine kinase receptors [6] prompted us to investigate whether RanBPM can interact with c-Kit as well (Figure 1A). HEK293 cells were transfected with plasmids encoding c-Kit and HA-tagged RanBPM (RanBPM-HA) and treated or not with the ligand SCF. Western blot analysis of lysates immunoprecipitated with an anti-Kit antibody revealed the presence of RanBPM by blotting with an anti-HA antibody. Since c-Kit protein is present in two forms, the mature 145 kDa form containing a complex glycosylation pattern and the precursor 125 kDa form containing a generic N-linked high-mannose oligosaccharide specific to the ER, we performed a reverse IP with the anti-HA antibody to investigate the specificity of RanBPM binding to these forms. Firstly, as shown in Figure 1A, we found that RanBPM can immunoprecipitate c-Kit, confirming the physical interaction between the two proteins. Secondly, we found that SCF stimulation did not affect the overall level of RanBPM and c-Kit interaction, since there was no change in the total amount of c-Kit pulled down by RanBPM before or after SCF treatment (Figure 1A). Treatment with the c-Kit inhibitor Dasatinib further confirmed that c-Kit binds RanBPM irrespective of its activation state since phosphorylation blockage in the presence of SCF did not influence the level of RanBPM/c-Kit interaction (Supplementary Figure S1). Importantly, the binding of RanBPM to the precursor 125 kDa c-Kit form strongly suggested that the two proteins already interact in the ER/Golgi before transport to the cell membrane and irrespective of c-Kit activation by SCF [14]. De-glycosylation treatment of HEK293 cells expressing c-Kit or of testis lysates, produced a single c-Kit immunoreactive band of about 100 kDa corresponding to the c-Kit native protein and confirming that c-Kit pulled down by RanBPM corresponds to proteins differentially glycosylated including a fully mature and a partially glycosylated one (data not shown [15]). This suggests that RanBPM can bind c-Kit during the maturation process in the ER/Golgi as well as the fully-mature, membrane bound receptor that signals upon SCF binding. The in vitro association led us to investigate whether RanBPM and c-Kit interact in vivo as well. To this end, we lysed testes from a 3-month old mouse, which express significant amounts of both RanBPM and c-Kit (see input Figure 1B) and performed immunoprecipitations with antibodies against c-Kit or RanBPM. We found that the anti-c-Kit antibodies could co-immunoprecipitate RanBPM and conversely anti-RanBPM antibodies could co-immunoprecipitate c-Kit, strongly supporting the physiological significance of a RanBPM interaction with c-Kit (Figure 1B, 1C).

To address which subdomains of RanBPM and c-Kit contribute to their interaction we performed IP experiments with constructs lacking specific protein domains (Figure 1D, 1E). Immunoprecipitation of RanBPM from deletion constructs showed that the SPRY domain is essential for the interaction of RanBPM with c-Kit (Figure 1D and Supplementary Figure S2). This result is not surprising considering that the SPRY domain is involved in protein/protein interactions [6]. Although deletion of any of the different regions of the c-kit intracellular domain lead to a general loss of c-kit interaction with RanBPM, the tyrosine kinase domains appeared to be mostly responsible for c-kit interaction to the RanBPM SPRY domain (Figure 1E and Supplementary Figure S2). Interestingly, RanBPM has been shown to interact in a similar fashion with the tyrosine kinase domain of the c-Met receptor through its SPRY domain [16].

### c-Kit protein levels and signaling are decreased in the testis of young *RanBPM*^−/−^ males

**Figure 2:**
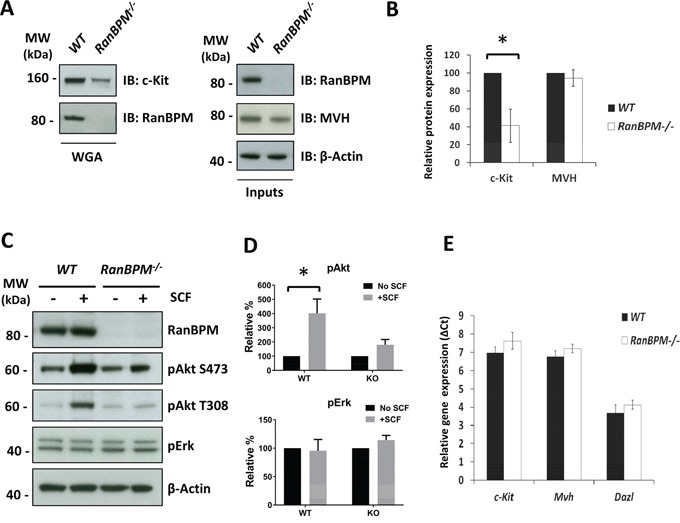
RanBPM affects c-Kit protein levels and signaling in the testis of young *RanBPM*^−/−^ mice **A.** c-Kit protein levels are decreased in the testis of young *RanBPM*^−/−^ mice. C-Kit protein was pulled-down using wheat germ agglutinin (WGA)-conjugated beads from postnatal day 9 (P9) testis lysates of *WT* or *RanBPM*^−/−^ mice (WGA lanes). Immunoblots (IB) were performed using anti-c-Kit and anti-RanBPM antibodies. MVH level in input lysates (right panels) was used as a marker of germ cells whereas β-actin was used as a loading control. **B.** quantification of c-Kit and MVH levels are shown as the mean ±SEM from three independent experiment as in A. **C.** c-Kit PI3K signaling is reduced in the testis of young *RanBPM*^−/−^ mice. Cells dissociated from P9 *WT* and *RanBPM*^−/−^ testes were stimulated or not with 100 ng/ml SCF for 15 minutes and, pAkt (both S473 and T308) and pErk levels were analyzed by Western blot. β-actin served as loading control. **D.** quantification of pAkt (S473) and pErk levels are shown as the mean ±SEM from three independent experiment as in C. **E.** *c-Kit* mRNA levels are unchanged in the testis of young *RanBPM*^−/−^ mice. Q-PCR analysis was performed using P9 *WT* and *RanBPM*^−/−^ testes and data are shown as the mean ±SEM from three independent experiments. Both MVH and Dazl were used as markers of germ cells. Molecular weight markers (MW) are indicated in kilo Dalton (kDa). * p<0.01.

The results from the interaction experiments prompted us to test whether RanBPM functionally affects c-kit in vivo (Figure 2). We first analyzed c-Kit protein levels in testis lysates from WT and *RanBPM*^−/−^ males at P9, a stage at which we first observed a reduction in spermatogonia proliferation in the mutant [11]. c-Kit expression in differentiating spermatogonia begins around P7 and its levels are still low at P9, to the extent that wheat germ agglutinin (WGA)-conjugated beads are required to successfully detect it by Western (Figure 2A, WGA lanes). Analysis of WGA pulled-down proteins shows a drastic decrease in c-Kit protein in the mutant testis. To test if this reduction is caused by a general decrease in germ cell number in the testis rather than a specific effect on c-Kit expression, we assessed the levels of Mouse Vasa Homolog (MVH), a marker for all types of germ cells, in the same lysates (Figure 2A, Inputs lanes, right panels) [17, 18]. Interestingly, MVH levels were not significantly different between WT and *RanBPM*^−/−^ testis, suggesting that c-Kit expression is specifically decreased in the mutant (Figure 2B). To confirm this finding we repeated this analysis at P8, a time point at which c-Kit level is even lower (Supplementary Figure S3A). Again we found a significant depletion of *c-kit* in the *RanBPM* mutant testis while MVH levels were completely unaffected by RanBPM loss (Supplementary Figure S3).

To investigate if the reduction of c-Kit protein levels in the RanBPM mutants had an effect on SCF signaling we dissociated P9 testicular cells from mutant and wild type littermates and stimulated them with SCF (Figure 2C). c-Kit signaling was assessed by looking at the phosphorylation of the downstream effectors Akt and Erk. Although it has been reported that both Erk and Akt are activated by SCF [19] we found that only Akt is phosphorylated in response to SCF (Figure 2C, 2D). This is most likely due to the fact that previous experiments had been performed at an earlier time point when there is still active spermatogonia proliferation, whereas at P9 most spermatogonia have differentiated [20]. Irrespective, our data indicated that the PI3-kinase pathway, which is crucial at this stage of spermatogenesis, was activated by the c-Kit ligand [12, 13] whereas in the RanBPM mutant mice SCF induced Akt phosphorylation levels were strikingly reduced compared to controls. These data suggest that c-Kit function in the testes may be the underlying cause of the loss of germ cells observed in RanBPM deleted testis (Figure 2C, 2D; Supplementary Figure S3).

Although the interaction between RanBPM and the mature c-Kit protein suggests a post-translational effect on c-Kit by RanBPM we also tested if RanBPM mutant testes had a defect in *c-kit* mRNA levels as well. Half of the mutant and control testes that were dissected for protein analysis were processed for RNA extraction and quantitative PCR analysis was performed to determine the relative mRNA level of *c-kit*. In addition to *Mvh, Dazl* (deleted in azoospermia-like), another gene involved in the differentiation of spermatogonia during the first wave of spermatogenesis [21], was also used as control. As shown in Figure 2E, no significant differences in the levels of *c-kit, Mvh* or *Dazl* mRNA levels were detected between WT and mutant testis, suggesting that the loss of RanBPM affects c-Kit protein levels post-transcriptionally.

### Silencing of RanBPM in erythroid-myeloid-lymphoid cells induces a decrease in c-Kit expression followed by cell death

**Figure 3:**
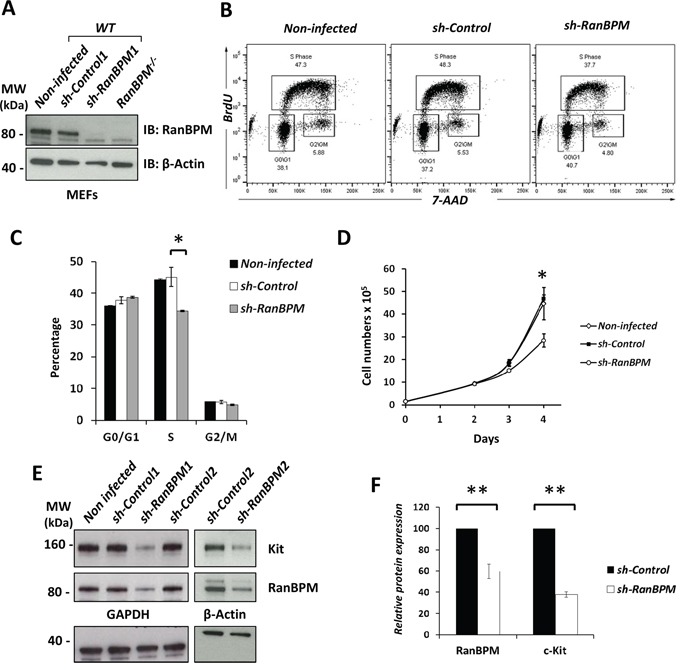
Silencing of RanBPM in EML cells causes a decrease in c-Kit levels and reduced cell growth **A.** sh-RNA for RanBPM is specific. Non-infected MEFs, MEFs infected with lentiviral vectors expressing *sh-Control1* or *sh-RanBPM1 RNAs* and MEFs isolated from *RanBPM*^−/−^ mice as control were analyzed by Western blot using an anti-RanBPM antibody. β-actin was used as a loading control. **B, C.** *RanBPM*-silenced EML cells have reduced cells in S phase. Representative flow cytometry graphs from EML cells infected with either sh-Control or sh-RanBPM constructs incubated with BrdU at day 3 after lentiviral infection and subsequent labeling with anti-BrdU antibodies and 7AAD (B). **C.** Bar graphs showing the percentage of cells in each phase of the cell cycle from cytometry analysis in B. **D.** growth curves showing reduced proliferation of EML cells infected with a *sh-RanBPM* lentiviral construct compared to non-infected or *sh-Control* infected cells. **E.** c-Kit protein levels are decreased in EML cells with silenced RanBPM. Protein lysates from non-infected EML cells and EML cells infected with 2 different *sh-RanBPM* or *sh-Control* constructs were analyzed by Western blot for c-Kit and RanBPM levels at day 3. GAPDH (bottom, left panel) and β-actin (bottom, right panel) served as loading controls. **F.** Bar graph showing quantification of RanBPM and c-Kit levels as the mean ±SEM from three independent experiments as in E. * p<0.05, **p<0.01.

To further investigate the specificity of RanBPM function on c-kit regulation and whether this role on its expression is present in other cell types we used cells that express c-Kit and are also dependent on its activation by SCF for proliferation and survival. Erythroid myeloid lymphoid (EML) cells are established multipotent hematopoietic cells that require SCF for survival in culture [22]. We reasoned that if RanBPM is required for c-Kit expression and/or function, its silencing would affect EML cell survival. To silence RanBPM we constructed lentiviral vectors and tested their specificity in mouse embryonic fibroblasts (MEFs). Compared to the *sh-Control*, the *sh-RanBPM* lentivirus was very effective in silencing RanBPM in MEFs as its protein expression was not detectable after infection (Figure 3A). However, the lentiviral vector was less effective in EML and required two consecutive infections to sufficiently silence RanBPM (Figure 3E). The day of the second infection was considered as day 1 and cells were counted thereafter for three consecutive days. No differences were observed in the number of cells on day 2 (Figure 3D). However, on day 3 we noticed a reduced growth rate of the EML cells with silenced RanBPM and this effect became even more striking by day 4, suggesting an effect on EML cell survival and/or proliferation (Figure 3D). To investigate the type of deficit we performed BrdU/7AAD labeling at day 3 followed by quantification of the cells in different phases of the cell cycle by flow cytometry (Figure 3B, 3C). While the cell-cycle profile of cells infected with a sh-Control construct showed no differences compared to non-infected cells, cells with a silenced RanBPM showed a significant reduction of cells in “S” phase (Figure 3B-3C). Moreover, when we subjected the cells to puromycin selection (the puro-resistant gene is part of the lentivirus vector) to eliminate cells that were not infected by the lentivirus, all cells in the *sh-RanBPM* infected samples died whereas the *sh-Control* cells displayed only modest cell death (data not shown). These data strongly suggest that RanBPM is required for EML viability.

To investigate whether silencing *RanBPM* caused c-Kit downregulation and consequent EML cell death, we analyzed c-Kit protein levels by Western blot at day 4 after lentiviral infection (Figure 3E). To further ensure the specificity of the action of the RanBPM-specific sh-RNA we employed a second *sh-RanBPM* construct (*sh-RanBPM2*) for the analysis. EML cells infected with either of the *sh-RanBPM* lentiviruses showed a significant decrease in c-Kit protein level, confirming that RanBPM is essential for normal c-Kit protein expression (Figure 3E, 3F).

### RanBPM regulation of c-Kit protein level in EML cells is specific

**Figure 4:**
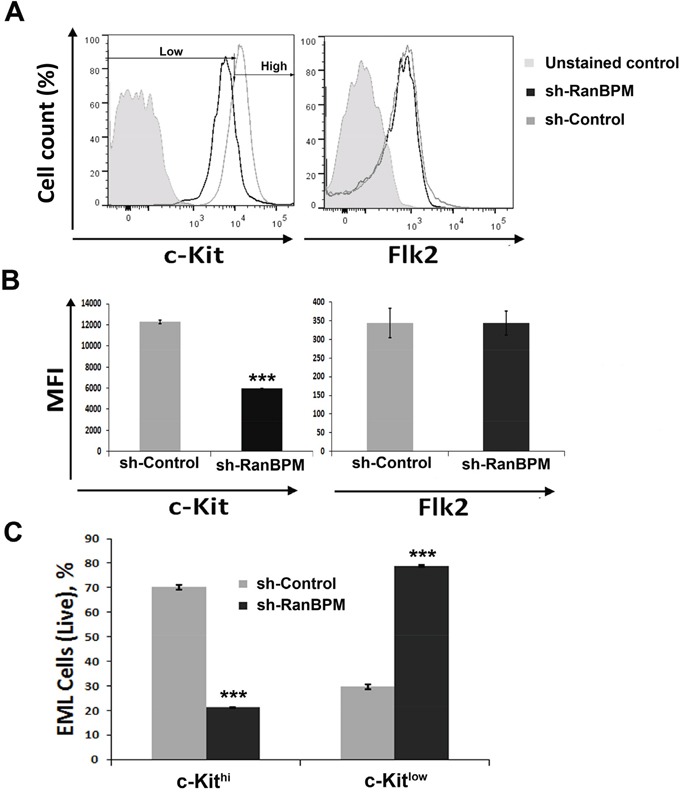
RanBPM silencing in EML cells causes specific downregulation of c-Kit **A.** Flow cytometric analysis of EML cells transduced with *RanBPM*-specific or *control shRNA* lentivirus showing the mean fluorescence intensity (MFI) of c-Kit (left panel) and Flk2 (right panel). Note the specific MFI reduction of c-Kit and not Flk2 in *sh-RanBPM* EML cells compared to *sh-Control* cells. **B.** quantification of the averages of c-kit and Flk2 MFI of EML cells transduced with *Control* or *RanBPM shRNA* from 3 independent experiments as in A. **C.** percentage of live EML cells with or without *RanBPM* knockdown expressing high or low levels of c-Kit in gated live cells. Data are presented as the mean ± S.E.M. and *** = *P*<0.001. C-kit intensity level was set as depicted in panel A (Low and High). Note the dramatic reduction in EML cells with high c-Kit membrane level after *sh-RanBPM* transduction.

Based on the data demonstrating that c-Kit down-regulation by RanBPM silencing in EML cells precedes their death, we tested whether other membrane bound receptors were affected by RanBPM expression (Figure 4). For this, we infected EML cells and, in addition to c-Kit, we examined the expression of other membrane bound receptors such as Flk2 (fetal liver kinase-2), a tyrosine kinase receptor expressed in hematopoietic progenitor cells that is important for their development and exhibits close structural similarities to c-Kit [23, 24]. In agreement with the Western results (Figure 3E), by FACS analysis we found that c-Kit receptor expression at the membrane was also reduced, while Flk-2 levels were similar in both *sh-Control* and *sh-RanBPM* infected cells (Figure 4A, 4B). Moreover, depletion of RanBPM caused an increase in the number of cells with low levels of c-Kit while cells with high levels of c-Kit were dramatically reduced (Figure 4C). Other membrane receptors including CD34 and CD48 were unchanged by RanBPM silencing, while the CD150 and stem cell antigen-1 (Sca-1) markers were only marginally affected (Supplementary Figure S4). Taken together, these data strongly suggest that RanBPM specifically controls c-Kit protein level in EML cells.

### Hematopoietic progenitor cell populations are affected in *RanBPM*^−/−^ mice

**Figure 5:**
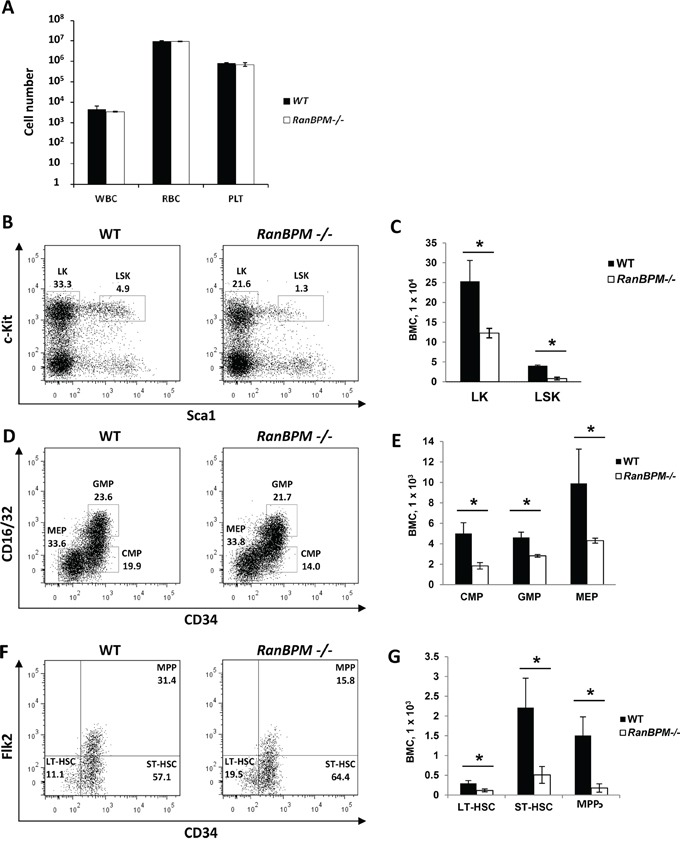
Hematopoietic progenitor cell populations are significantly reduced in *RanBPM*^−/−^ mice **A.** Absolute number of platelets (PLT), white (WBC) and red (RBC) blood cells in control and *RanBPM*^−/−^ mice (number/uL). **B-G.** Analysis of hematopoietic stem and progenitor cell populations in the bone marrow of WT and *RanBPM*^−/−^ mice. Representative flow cytometric analyses of Lineage-negative cells (B), LK cells (D) and LSK cells (F) from the bone marrow of WT and *RanBPM*^−/−^ mice. The gates used to distinguish the different populations together with the specific total number of cells are displayed. C, E and G: quantification of gated cells respectively from B, D and F; note, in all graphs, the significant reduction in all lineages total number± SEM (*n* = 3 mice per genotype) of different c-kit progenitor cells. * p<0.05. Cells were incubated with antibodies that recognize lineage specific cell surface antigens (Mac-1, Gr-1, B220, Ter119, CD4, CD8, and IL-7Rα) and antibodies to distinguish HSPC including PE-conjugated c-Kit, APC-conjugated Sca-1, FITC–conjugated CD34, PE-Cy5-conjugated Flk2 (Flt3), and PE-Cy7-conjugated FcγRII/III. LK, lineage-negative c-kit^+^/Sca1^−^ (LK) progenitors; LSK, lineage negative c-Kit^+^/Sca1^+^; MPP, multi-potent progenitors; CMP, common myeloid progenitors; GMP, granulocyte macrophage progenitors; MEP, megakaryocyte erythrocyte progenitors.

SCF and c-Kit play important roles not only in gametogenesis but also in hematopoiesis [1]. Mutations in these genes cause anemia in mice due to defects in progenitor cells of the hematopoietic lineage [1, 25]. The finding that RanBPM is essential for normal c-Kit protein expression in cells of the hematopoietic system lineage (EML cells) prompted us to investigate whether *RanBPM* mutant mice also had hematopoietic defects in addition to the sterility phenotype. A complete blood count cell analysis of peripheral blood from age matched WT and *RanBPM*^−/−^ mice showed no differences in the number of white blood cells, red blood cells and platelets (Figure 5A). However, analysis of *RanBPM*^−/−^ bone marrow progenitors showed a decrease in the percentage and total number of committed lineage-negative c-kit^+^/Sca1^−^ (LK) progenitors, and lineage negative c-Kit^+^/Sca1^+^ (LSK; Figure 5B, 5C) cells that contain multi-potent progenitors (MPPs) and hematopoietic stem cells (HSCs) (Figure 5B-5G, and Supplementary Figure S5B). Reduction in LSK cells in *RanBPM*^−/−^ mice was the result of decreased total numbers of MPP (LSK Flk2^+^-CD34^+^), long-term-HSC (LT-HSC; LSK Flk2^−^ CD34^−^) and short-term (ST)-HSC (ST-HSC; Flk2^−^-CD34^+^) (Figure 5F, 5G, and Supplementary Figure 5D). While the percentage of LK subpopulations including common myeloid progenitors (CMP; CD16/32^−^-CD34^+^), granulocyte macrophage GMP (GMP; CD16/32^+^-CD34^+^) and megakaryocyte erythrocyte (MEP; CD16/32^−^-CD34^−^) progenitors were not significantly affected (Supplementary Figure S5C), the total numbers of CMP, GMP and MEP cells were significantly reduced (Figure 5D, 5E). Altogether, these data suggest that loss of RanBPM expression impairs normal hematopoietic development in the bone marrow, which results in reduced numbers of all HSPC populations that express c-Kit *in vivo*.

The defects observed in the *RanBPM* deficient mice, are less dramatic than the ones observed by deletion of *c-kit* [2, 3] suggesting that (a) RanBPM is only part of the complex machinery regulating *c-kit* expression, or (b) other compensatory molecules can be at play. *RanBP10* is a gene highly homologous to *RanBPM.* Most importantly, its SPRY domain is almost identical to the RanBPM SPRY domain not only in terms of DNA sequence, but also structurally [26]. To test whether RanBP10 expression is affected by RanBPM loss in a physiological system, we evaluated the level of expression of RanBP10 in RanBPM deficient embryonic stem (ES) cells [11]. We found that complete loss of RanBPM causes upregulation of RanBP10 suggesting a direct relationship between the expression of the two genes (Supplementary Figure S6). Importantly, transfection of a *c-kit* expressing vector showed that c-kit levels were similar in control or *RanBPM* deficient ES cells with upregulated RanBP10 (Supplementary Figure S6). These data, coupled with the virtually identical SPRY domain in RanBPM and RanBP10, suggest that RanBP10 can potentially compensate for RanBPM loss.

### RanBPM regulates c-Kit level in human cells

**Figure 6:**
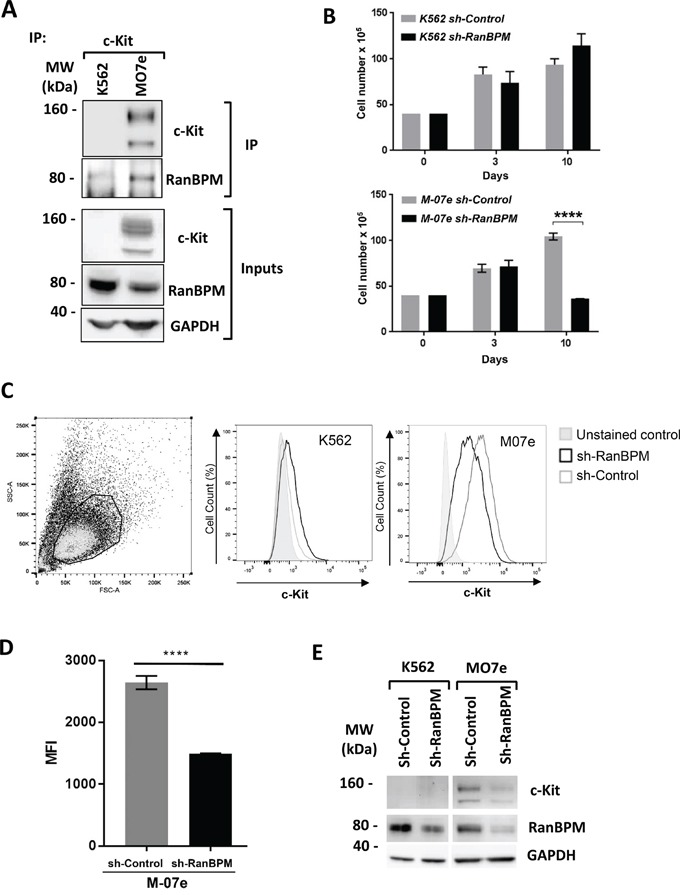
RanBPM regulation of c-kit level influences human cell lines growth **A.** c-Kit binds to RanBPM in the M07e human myeloid leukemia cell line. M07e and K562 cell lysates were immunoprecipitated with an anti-c-Kit antibody and blotted with the indicated antibodies. Note the presence of RanBPM pulled down by c-Kit in the M07e cells. Inputs represent 10% of the protein lysates used for the IP experiment. MW, molecular weight markers in kDa. **B.** Total cellularity of M07e and K562 cultures following transduction with *sh-Control* or *sh-Ran*BPM lentiviruses. Cells were transduced on day 0, and puromycin treated (1 ug/mL) on day 3 and continuously thereafter. Error bars indicate total cell number +/− SEM. **C.** Representative dot plot showing forward and side scatter gating for live cells. Bold lines indicate the live gate, excluding dead and dying cells (left panel). In central and right panels are the representative flow cytometry histograms showing c-Kit expression (indicated by anti-c-Kit-PE fluorescence) on live-gated M07e or K562 cells. **D.** mean fluorescence intensity (MFI) of c-Kit-PE-stained cells for live-gated M07e cells. Cells were transduced with either sh-Control or sh-RanBPM lentiviruses as described above. Data are from cultures analyzed at day 10. Error bars represent mean fluorescence intensity +/− SEM. **** P<0.0001. **E.** Western blot analysis of protein lysates from the K562 and M07e cells at day 10 from transduction with the *sh-Control* or *sh-RanBPM RNA* as in B-D. Blots were hybridized with c-Kit, RanBPM and GAPDH specific antibodies as indicated. Note the effectiveness of the *Sh-RanBPM-specific RNA* in downregulating RanBPM in both K562 and M07e cells and the reduction in c-Kit expression in the M07e cells.

The finding that in mouse depletion of RanBPM causes c-Kit downregulation in cells of the reproductive and hematopoietic system prompted us to investigate whether this regulatory mechanism is present in human cells as well. For this we used two human myeloid leukemia cell lines, M07e cells [27], which endogenously express wild-type c-Kit and are dependent on SCF, and K562 cells, which are c-Kit negative. Both lines express RanBPM (Figure 6A, inputs). Importantly, when M07e and K562 cell lysates were subjected to IP experiments with an anti-c-kit antibody, RanBPM was readily pulled-down in the M07e cells suggesting that these two proteins interact in human cells. It also should be noted that c-Kit and RanBPM in M07e cells are expressed endogenously and not in a transfected overexpression context further validating the physiological significance of this interaction. Next, we explored whether RanBPM regulates c-Kit level in human cells by transducing M07e and K562 cells with the *sh-Control* and *sh-RanBPM*-specific lentiviruses and by studying their growth rate and c-Kit expression level by FACS analysis (Figure 6B-6E). M07e cells transduced with the *sh-RanBPM* lentivirus displayed blunted growth (Figure 6B) similar to what was observed in EML cells. Importantly, this lentivirus had no effect on the growth of the c-Kit negative K562 cells. Furthermore, FACS analysis showed a specific reduction in c-Kit membrane expression levels in the M07e cells transduced with the *sh-RanBPM* lentivirus and not in the *sh-Control* sample (Figure 6C, 6D). Together these data strongly suggest that RanBPM regulates c-Kit levels in human cells.

## DISCUSSION

Elucidating the cellular network that controls receptor tyrosine kinase activity is key to understanding how they regulate cellular processes including cell proliferation and survival, cell metabolism, cell death, cell migration and differentiation [28, 29]. The tyrosine kinase receptor c-Kit is critical for normal development of germ cells, pigment cells and hematopoietic cells [2, 3]. A number of studies have shown that its function is regulated at the transcriptional and translational levels but can also be dictated by its interaction with proteins expressed in specific cell types or subcellular compartments [3, 30–33]. The identification of mechanisms that control not only its presence or absence in a cell, but also its relative level, may be relevant to the understanding of the self-renewal properties of HSCs [5]. This study identifies RanPBM as a new scaffold protein regulating c-Kit expression in the testis and in specific cell lineages of the mouse immune system. These data are supported by the in vitro and in vivo findings that RanBPM deletion causes loss of c-Kit expression preceding cell death in cell types that are dependent on c-Kit signaling, such as differentiating spermatogonia or EML cells, and RanBPM knockout mice have defects in hematopoietic lineage cells expressing c-Kit. Importantly, this c-Kit regulatory mechanism is also present in human cells expressing these genes endogenously.

An as yet unresolved question is why the loss of RanBPM does not cause obvious deficits in other cell types expressing c-Kit such as melanocytes and mast cells, or a more profound phenotype in progenitor cells of the hematopoietic lineage. In addition to RanBPM, mammals encode another scaffold protein, RanBP10 that shares significant sequence similarities with RanBPM and is expressed in the spleen and bone marrow [34]. Except for lacking the N-terminal proline-rich domain, RanBP10 possesses all other RanBPM domains which are very conserved between the two proteins [35]. Importantly, it has been reported that the SPRY domain, which we found to be responsible for the binding of RanBPM to c-Kit (Figure 1D), is extremely conserved between RanBPM and RanBP10 not only in terms of sequence, but also structurally [26] and both RanBPM and RanBP10 bind to the tyrosine kinase domain of the c-met receptor [16]. Our finding that in ES cells lacking RanBPM, RanBP10 is upregulated and the observation that mice lacking both RanBPM and RanBP10 die in utero around day 13 of gestation (SP and LT unpublished results) while the individual knockout mice are viable [11, 36], support the notion that these proteins can exert overlapping functions. In future studies, it will be important to investigate whether RanBPM deficiency causes a phenotype only in some cell populations because they lack mechanisms to up-regulate RanBP10, or because RanBP10 can rescue only specific RanBPM functions.

The *c-kit* gene is mutated in a number of malignancies including leukemia, tumors of the gastrointestinal tract and germ cells where it is rendered constitutively active by mutations in the juxtamembrane or kinase domain. Our study is important because it unveils a new mechanism regulating c-Kit expression potentially useful in harnessing oncogenic c-Kit [2]. The finding that RanBPM can bind both the mature and immature, partially glycosylated c-Kit form irrespective of its binding to SCF suggests that blocking the RanBPM SPRY domain may provide a general strategy to treat different oncogenic c-Kit variants. This is particularly important because oncogenic c-Kit signaling can occur from different intracellular compartments, with mutations altering both its trafficking and activation [2, 31]. Further studies should focus on investigating whether blocking RanBPM function can control not only wild-type but also oncogenic c-Kit.

In addition to c-Kit, RanBPM binds other tyrosine kinase receptors including the HGF receptor Met, the Gas6 receptor Axl, and the neurotrophin receptors TrkA and TrkB [37–40]. However, phenotypes caused by RanBPM deficiency have not yet been linked to a direct defect in signaling regulation of these receptors. Thus, one of the most exciting findings of this study is the establishment of a link between loss of RanBPM and downregulation of c-Kit expression, and developmental defects that phenocopy, at least in part, loss of c-Kit function [1, 11–13]. Our data also suggest that RanBPM may not have a general role in the control of c-kit expression but a more specific one depending on cellular context or may have specific roles that can not be compensated by RanBP10. The high level of specificity of RanBPM in the control of c-Kit function is also suggested by the fact that its downregulation in EML cells does not change the expression of Flk-2, a tyrosine kinase receptor belonging to the same class of receptors as c-Kit [28]. This is an important finding because it suggests that scaffold proteins can be part of the cellular mechanism used to control and fine tune downstream pathways activated by tyrosine kinase receptors. For example, in PC12 cells, RanBPM modulates BDNF-induced neuronal morphogenesis by regulating TrkB activation of MAPK and Akt [38], and, in human renal carcinoma cells, RanBPM enhances HGF-induced cell migration by interacting with Met and promoting GTP-Ras association and Erk phosphorylation [40].

Here we found that RanBPM binds to the precursor 125 kDa c-Kit form, which is present exclusively in the ER/Golgi [41], suggesting that the two proteins already interact in the ER before transport to the cell membrane. This finding suggests that RanBPM may have a dual role; it may have a chaperone function during the maturation process of the c-Kit protein in the ER, and a scaffold function at the membrane during c-Kit activation by SCF. In this regard, it would be interesting to investigate whether RanBPM is involved in c-Kit recruitment to lipid rafts where it is required for the efficient activation of the PI3-K/Akt pathway and proliferation [42]. Such a scaffolding role may also be at work for other membrane proteins. For example, both RanBP9 and 10 interact with protein kinase C (PKC) γ and δ and the D_1_ dopamine receptors but do not seem to modulate the kinase activities of PKCδ or PKCγ. Rather, they appear to regulate the spatial and temporal organization of the D_1_ receptor-PKC signaling complex [43] supporting the notion that one of the main activities of RanBP9 relates to its scaffolding function as an organizer of protein complexes involved in trafficking and signaling. If this is the case, and RanBPM influences c-Kit signaling by controlling its subcellular spatial organization, targeting the interaction of these two proteins may provide a new modality to affect c-Kit signaling in malignancies or long-term maintenance and expansion of hematopoietic stem and progenitor cells [2, 4].

The multiple scaffolding roles of RanBPM and the many proteins to which it binds makes it a challenge to identify how it exerts such a variety of biological functions. Nevertheless, the identification of proteins such as c-Kit that are directly affected by RanBPM in controlling specific physiological functions is an important step towards filling this gap. In relation to c-Kit function, the identification of a new regulatory molecule may provide a new tool toward the understanding of how to harness the activity of this tyrosine kinase receptor important for oncogenesis and regenerative medicine [44].

## MATERIALS AND METHODS

### Mice

*RanBPM*^−/−^ mice, previously reported [11] were group-housed under standard conditions with food and water available ad libitum. All experiments were performed in compliance with the NIH guidelines for animal care and use of experimental animals.

### Cell culture

Primary mouse embryonic fibroblasts MEFs were generated and cultured according to standard protocols [45]. The Erythroid Myeloid Lymphoid (EML) cell line described previously [22] was maintained in Iscove's Modified Dulbecco's Medium (IMDM), 20% fetal bovine serum (FBS), Glutamax, penicillin-streptomycin supplemented with 100 ng/mL recombinant murine SCF (Peprotech). Human cell lines K562 [46] and M07e [27] were maintained in RPMI-1640 with glutamine and HEPES (Invitrogen), 10% FBS, penicillin-streptomycin. M07e were supplemented with 100 ng/mL recombinant human SCF and 30 ng/mL GM-CSF (Peprotech).

### Isolation of male germ cells

Postnatal male germ cells were isolated from P9 WT and *RanBPM*^−/−^ mice as previously described [11].

### Transfection

HEK293 cells were transfected with 3 μg of pcDNA 3.1 (Invitrogen)-RanBPM-HA tagged and 3 μg of pcDNA 3.1-c-Kit plasmids using the X-tremeGENE 9 DNA transfection reagent according to manufacturer's instructions (Roche).

### Immunoprecipitation, wheat germ agglutination (WGA) and western analysis

HEK293, K562 and M0E7 cells and P9 or 3 month-old mouse testes were homogenized in 1X RIPA buffer (Millipore) supplemented with protease inhibitors (complete mini, Roche) with rotation at 4°C for 30 min. Cell lysates were centrifuged at 15,000 g for 20 min at 4°C. Cell debris were discarded, protein amount quantified and co-immunoprecipitations were performed overnight at 4°C using anti-c-Kit (Santa Cruz) or anti-HA antibodies (Abcam) and specific Ig isotypes as negative controls. Pre-blocked protein G beads were used to pull-down the protein complexes (Trueblot).

WGA pull-down experiments were performed as previously reported [47]. Antibodies were: goat anti-c-Kit (Santa Cruz; R&D) for mouse experiments; anti-c-kit mouse monoclonal antibody (E-3) against the c-terminus of human c-kit (Santa Cruz) for the human line experiments, Phospho-c-kit (Tyr703; Cell Signaling), rabbit anti-RanBPM (Abcam), anti-HA-Peroxidase high affinity (Roche), anti-pAkt, anti-pErk (Cell signaling), anti-β-actin-HRP, anti-MVH (Abcam), anti-GAPDH mouse monoclonal against rabbit muscle GAPDH (Millipore, MAB374).

### Q-PCR

Total RNA was extracted from P9 WT and RanBPM^−/−^ mouse testes using an RNeasy mini kit (Qiagen) and treated with DNaseI (Roche). First-strand cDNA was generated by reverse transcription of 1 μg of RNA using the Superscript III first-strand synthesis system (Invitrogen) and random hexamer nucleotides.

Quantitative PCR was performed with an MX4000 apparatus and software (Stratagene) using iTaq Universal SYBR Green Supermix (BioRad). Primer sets for each sequence analyzed were:

c-Kit 5'-GATCTGCTCTGCGTCCTGTT-3' and 3'-CTGATTGTGCTGGATGGATG-5'; MVH 5'-ACC AAGATCAGGGGACACAG-3' and 3'-TAACC ACCTCGACCACTTCC-5'; Dazl 5'-ATGTCT GCCACAACTTCTGAG-3' and 3'-CTGATTTC GGTTTCATCCATCCT-5'; β-Actin 5'-GACGGCC AGGTCATCACTAT-3' and 3'-ATGCCACAGG ATTCCATACC-5'. Primers were designed to span large introns and produce a single DNA band by gel electrophoresis without dimerization products as assessed by melting point analysis on the MX4000. Conditions for amplification were: one cycle of 95°C for 3 min followed by 40 cycles of 95°C, 10 sec, 60°C, 20 sec. Quantitative PCR reactions were performed in triplicate and cycle numbers were averaged. Gene expression was normalized to that of β-Actin.

### Silencing of RanBPM expression, FACS analysis

Lentivirus production was carried out by standard technique from two independent controls (Sh-Control1 and sh-Control2) and RanBPM Sh-RNA (TRCN0000102112 and TRCN0000102113 from Open Biosystems). Lentiviral vectors included a puromycin-resistance cassette for selection of infected cells. After 24 hours of incubation, MEFs were re-infected with the same amount of the virus and after 3 days cells were lysed and processed for immunoblotting. For knock-down of RanBPM in EML cells, 0.1 x 10^6^ EML cells/well were plated in a 6-well-plate and lentivirus infected. After 24 hours, cells were infected again with the same amount of virus (considered Day 1). On day 2, fresh medium was added to the cultures and cell counting performed every 24 hours for 3 days using an automated cell counter (Nexcelom Bioscience). For transduction of human cells, method was as described above, except 4 x10^6^ cells per well were plated at Day 0. One ug/mL puromycin (Invitrogen) was added at day 3 and maintained in culture thereafter.

### Mouse bone marrow, EML and human cell line immunophenotype analysis

To quantify HSPC, we purified mononuclear cells from bone marrow using lymphocyte separation medium (LSM) (MP Biomedicals). Flow cytometry was performed with a BD LSRII (Becton Dickenson) and data was analyzed with FlowJo software (Treestar). BrdU-7AAD labeling of EML cells was performed with a BrdU/7AAD kit (BD Pharmingen) at day 3 after lentiviral infection followed by flow cytometry analysis to quantify cells at different cell cycle phases. For human cell lines, cells were labeled with anti-human c-Kit-PE (Clone 104D2, eBioscience) as per the manufacturer's instructions and analyzed as described above.

### Cellular blood counts

Cellular blood counts (CBC) were determined by analyzing 50 uL of peripheral blood in a HemaVet blood counter (CDC Technologies).

## SUPPLEMENTARY MATERIALS FIGURES



## References

[1] Morrison-Graham K, Takahashi Y (1993). Steel factor and c-kit receptor: from mutants to a growth factor system. BioEssays.

[2] Lennartsson J, Ronnstrand L (2012). Stem cell factor receptor/c-Kit: from basic science to clinical implications. Physiological reviews.

[3] Rojas-Sutterlin S, Lecuyer E, Hoang T (2014). Kit and Scl regulation of hematopoietic stem cells. Curr Opin Hematol.

[4] Kimura Y, Ding B, Imai N, Nolan DJ, Butler JM, Rafii S (2011). c-Kit-mediated functional positioning of stem cells to their niches is essential for maintenance and regeneration of adult hematopoiesis. PloS one.

[5] Shin JY, Hu W, Naramura M, Park CY (2014). High c-Kit expression identifies hematopoietic stem cells with impaired self-renewal and megakaryocytic bias. The Journal of experimental medicine.

[6] Suresh B, Ramakrishna S, Baek KH (2012). Diverse roles of the scaffolding protein RanBPM. Drug discovery today.

[7] Murrin LC, Talbot JN (2007). RanBPM, a scaffolding protein in the immune and nervous systems. Journal of neuroimmune pharmacology.

[8] Puverel S, Tessarollo L (2013). RanBPM, a scaffolding protein for gametogenesis. Current topics in developmental biology.

[9] Lakshmana MK, Yoon IS, Chen E, Bianchi E, Koo EH, Kang DE (2009). Novel role of RanBP9 in BACE1 processing of amyloid precursor protein and amyloid beta peptide generation. The Journal of biological chemistry.

[10] Woo JA, Jung AR, Lakshmana MK, Bedrossian A, Lim Y, Bu JH, Park SA, Koo EH, Mook-Jung I, Kang DE (2012). Pivotal role of the RanBP9-cofilin pathway in Abeta-induced apoptosis and neurodegeneration. Cell death and differentiation.

[11] Puverel S, Barrick C, Dolci S, Coppola V, Tessarollo L (2011). RanBPM is essential for mouse spermatogenesis and oogenesis. Development.

[12] Blume-Jensen P, Jiang G, Hyman R, Lee KF, O'Gorman S, Hunter T (2000). Kit/stem cell factor receptor-induced activation of phosphatidylinositol 3'-kinase is essential for male fertility. Nature genetics.

[13] Kissel H, Timokhina I, Hardy MP, Rothschild G, Tajima Y, Soares V, Angeles M, Whitlow SR, Manova K, Besmer P (2000). Point mutation in kit receptor tyrosine kinase reveals essential roles for kit signaling in spermatogenesis and oogenesis without affecting other kit responses. The EMBO journal.

[14] Brahimi-Adouane S, Bachet JB, Tabone-Eglinger S, Subra F, Capron C, Blay JY, Emile JF (2013). Effects of endoplasmic reticulum stressors on maturation and signaling of hemizygous and heterozygous wild-type and mutant forms of KIT. Mol Oncol.

[15] Bougherara H, Subra F, Crepin R, Tauc P, Auclair C, Poul MA (2009). The aberrant localization of oncogenic kit tyrosine kinase receptor mutants is reversed on specific inhibitory treatment. Mol Cancer Res.

[16] Wang D, Li Z, Schoen SR, Messing EM, Wu G (2004). A novel MET-interacting protein shares high sequence similarity with RanBPM, but fails to stimulate MET-induced Ras/Erk signaling. Biochemical and biophysical research communications.

[17] Fujiwara Y, Komiya T, Kawabata H, Sato M, Fujimoto H, Furusawa M, Noce T (1994). Isolation of a DEAD-family protein gene that encodes a murine homolog of Drosophila vasa and its specific expression in germ cell lineage. Proceedings of the National Academy of Sciences of the United States of America.

[18] Toyooka Y, Tsunekawa N, Takahashi Y, Matsui Y, Satoh M, Noce T (2000). Expression and intracellular localization of mouse Vasa-homologue protein during germ cell development. Mechanisms of development.

[19] Dolci S, Pellegrini M, Di Agostino S, Geremia R, Rossi P (2001). Signaling through extracellular signal-regulated kinase is required for spermatogonial proliferative response to stem cell factor. The Journal of biological chemistry.

[20] Bellve AR, Cavicchia JC, Millette CF, O'Brien DA, Bhatnagar YM, Dym M (1977). Spermatogenic cells of the prepuberal mouse. Isolation and morphological characterization. The Journal of cell biology.

[21] Schrans-Stassen BH, Saunders PT, Cooke HJ, de Rooij DG (2001). Nature of the spermatogenic arrest in Dazl −/− mice. Biology of reproduction.

[22] Tsai S, Bartelmez S, Sitnicka E, Collins S (1994). Lymphohematopoietic progenitors immortalized by a retroviral vector harboring a dominant-negative retinoic acid receptor can recapitulate lymphoid, myeloid, and erythroid development. Genes & development.

[23] Matthews W, Jordan CT, Wiegand GW, Pardoll D, Lemischka IR (1991). A receptor tyrosine kinase specific to hematopoietic stem and progenitor cell-enriched populations. Cell.

[24] Christensen JL, Weissman IL (2001). Flk-2 is a marker in hematopoietic stem cell differentiation: a simple method to isolate long-term stem cells. Proceedings of the National Academy of Sciences of the United States of America.

[25] Geissler EN, McFarland EC, Russell ES (1981). Analysis of pleiotropism at the dominant white-spotting (W) locus of the house mouse: a description of ten new W alleles. Genetics.

[26] Hong SK, Kim KH, Song EJ, Kim EE (2016). Structural Basis for the Interaction between the IUS-SPRY Domain of RanBPM and DDX-4 in Germ Cell Development. J Mol Biol.

[27] Kuriu A, Ikeda H, Kanakura Y, Griffin JD, Druker B, Yagura H, Kitayama H, Ishikawa J, Nishiura T, Kanayama Y (1991). Proliferation of human myeloid leukemia cell line associated with the tyrosine-phosphorylation and activation of the proto-oncogene c-kit product. Blood.

[28] Lemmon MA, Schlessinger J (2010). Cell signaling by receptor tyrosine kinases. Cell.

[29] Mehlen P (2010). Dependence receptors: the trophic theory revisited. Science signaling.

[30] Cruse G, Beaven MA, Music SC, Bradding P, Gilfillan AM, Metcalfe DD (2015). The CD20 homologue MS4A4 directs trafficking of KIT toward clathrin-independent endocytosis pathways and thus regulates receptor signaling and recycling. Molecular biology of the cell.

[31] Obata Y, Toyoshima S, Wakamatsu E, Suzuki S, Ogawa S, Esumi H, Abe R (2014). Oncogenic Kit signals on endolysosomes and endoplasmic reticulum are essential for neoplastic mast cell proliferation. Nature communications.

[32] Rossi P (2013). Transcriptional control of KIT gene expression during germ cell development. The International journal of developmental biology.

[33] Masson K, Heiss E, Band H, Ronnstrand L (2006). Direct binding of Cbl to Tyr568 and Tyr936 of the stem cell factor receptor/c-Kit is required for ligand-induced ubiquitination, internalization and degradation. The Biochemical journal.

[34] Schulze H, Dose M, Korpal M, Meyer I, Italiano JE, Shivdasani RA (2008). RanBP10 is a cytoplasmic guanine nucleotide exchange factor that modulates noncentrosomal microtubules. The Journal of biological chemistry.

[35] Yudin D, Fainzilber M (2009). Ran on tracks-cytoplasmic roles for a nuclear regulator. Journal of cell science.

[36] Kunert S, Meyer I, Fleischhauer S, Wannack M, Fiedler J, Shivdasani RA, Schulze H (2009). The microtubule modulator RanBP10 plays a critical role in regulation of platelet discoid shape and degranulation. Blood.

[37] Hafizi S, Gustafsson A, Stenhoff J, Dahlback B (2005). The Ran binding protein RanBPM interacts with Axl and Sky receptor tyrosine kinases. The international journal of biochemistry & cell biology.

[38] Yin YX, Sun ZP, Huang SH, Zhao L, Geng Z, Chen ZY (2010). RanBPM contributes to TrkB signaling and regulates brain-derived neurotrophic factor-induced neuronal morphogenesis and survival. Journal of neurochemistry.

[39] Yuan Y, Fu C, Chen H, Wang X, Deng W, Huang BR (2006). The Ran binding protein RanBPM interacts with TrkA receptor. Neuroscience letters.

[40] Wang D, Li Z, Messing EM, Wu G (2002). Activation of Ras/Erk pathway by a novel MET-interacting protein RanBPM. The Journal of biological chemistry.

[41] Shi H, Drummond CA, Fan X, Haller ST, Liu J, Malhotra D, Tian J (2016). Hiding inside? Intracellular expression of non-glycosylated c-kit protein in cardiac progenitor cells. Stem Cell Res.

[42] Jahn T, Leifheit E, Gooch S, Sindhu S, Weinberg K (2007). Lipid rafts are required for Kit survival and proliferation signals. Blood.

[43] Rex EB, Rankin ML, Yang Y, Lu Q, Gerfen CR, Jose PA, Sibley DR (2010). Identification of RanBP 9/10 as interacting partners for protein kinase C (PKC) gamma/delta and the D1 dopamine receptor: regulation of PKC-mediated receptor phosphorylation. Molecular pharmacology.

[44] Keith MC, Bolli R (2015). “String theory” of c-kit(pos) cardiac cells: a new paradigm regarding the nature of these cells that may reconcile apparently discrepant results. Circulation research.

[45] Southon E, Tessarollo L (2009). Manipulating mouse embryonic stem cells. Methods Mol Biol.

[46] Lozzio BB, Lozzio CB (1977). Properties of the K562 cell line derived from a patient with chronic myeloid leukemia. Int J Cancer.

[47] Fulgenzi G, Tomassoni-Ardori F, Babini L, Becker J, Barrick C, Puverel S, Tessarollo L (2015). BDNF modulates heart contraction force and long-term homeostasis through truncated TrkB. T1 receptor activation. The Journal of cell biology.

[48] Salemi LM, Loureiro SO, Schild-Poulter C (2015). Characterization of RanBPM molecular determinants that control its subcellular localization. PloS one.

