# Supplementary material for: RanBPM (RanBP9) regulates mouse c-Kit receptor level and is essential for normal development of bone marrow progenitor cells
Source: Oncotarget. 2016 Nov 8;7(51):85109–23. doi: 10.18632/oncotarget.13198 (PMC5341297; doi:10.18632/oncotarget.13198)
Supplement: Supplementary file 1 [file oncotarget-07-85109-s001.pdf]

RanBPM (RanBP9) regulates mouse c-Kit receptor level and is essential for normal development of bone marrow progenitor cells

SUPPLEMENTARY FIGURES

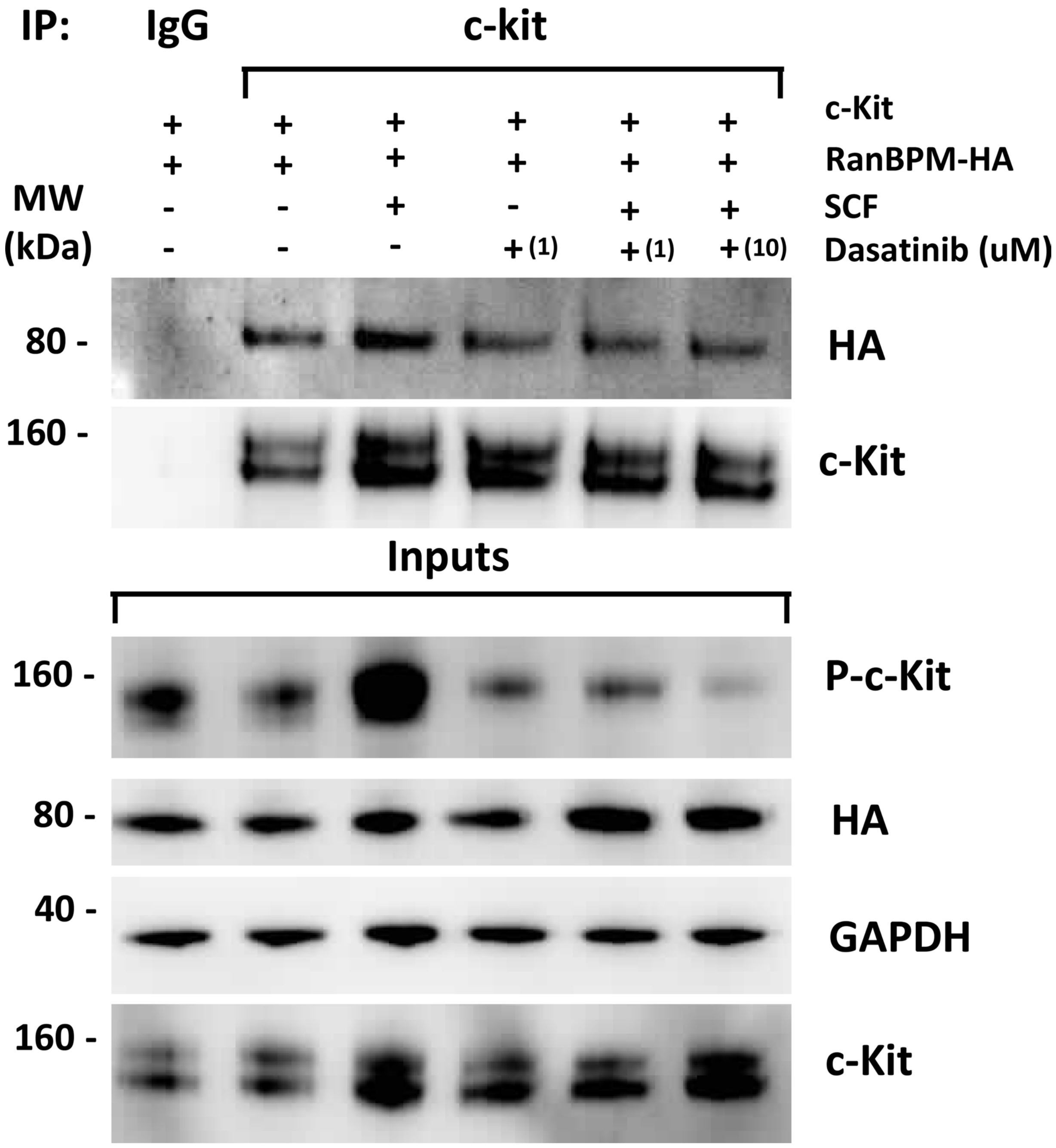

Supplementary Figure S1: The tyrosine kinase inhibitor Dasatinib does not block the binding of RanBPM to c-kit. Protein lysates from HEK293 cells transfected with RanBPM-HA and c-Kit cDNAs, were treated with the indicated dose of Dasatinib and stimulated or not with 100 ng/mL SCF for 10' before lysis and immunoprecipitation (IP) with an anti-c-kit antibody or a goat serum isotype as control (IgG). Western blots were performed with the antibodies indicated on the right (anti-HA; c-kit, GAPDH and anti Phospho-c-kit. Note the blocking of c-kit phosphorylation (P-c-Kit) by SCF in the presence of Dasatinib.

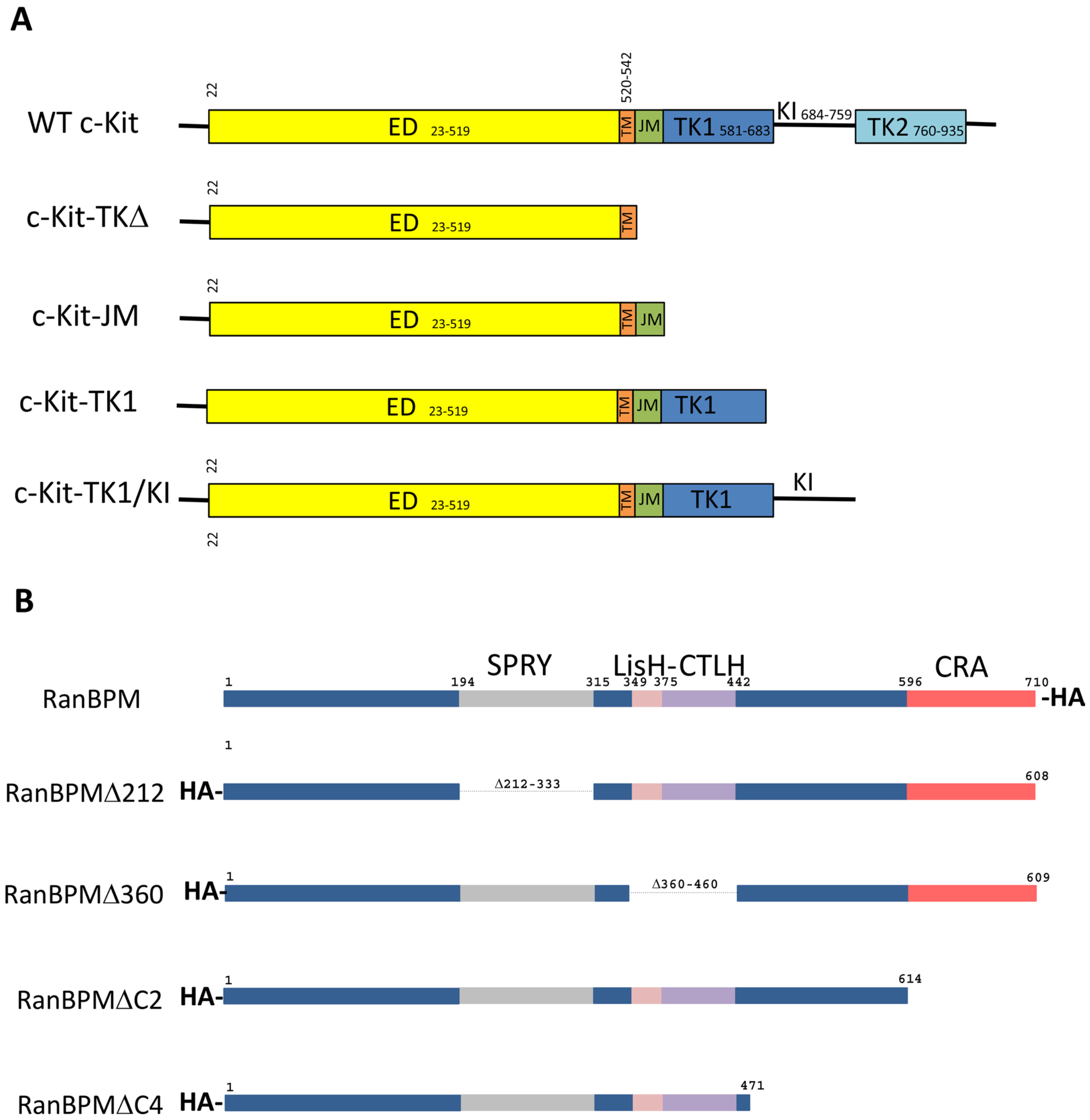

Supplementary Figure S2: Schematic representation of the c-kit and RanBPM mutant constructs used to identify the domains mediating their interaction. A. WT, wild type c-Kit; c-Kit-TK Δ, construct lacking both tyrosine kinase domains (TK1 and TK2) and the intracellular juxtamembrane region (JM); c-Kit-JM, construct with only the JM domain; c-Kit-TK1, construct having the JM and TK1 domain; c-Kit-TK1/KI, construct lacking only the TK2 domain but retaining the kinase intradomain (KI). **B.** RanBPM constructs were as previously described [48]. RanBPM; wild type RanBPM tagged with the HA domain. Deletions are as depicted in the schematic with the SPRY, LisH/CTLH and the CRA domains highlighted.

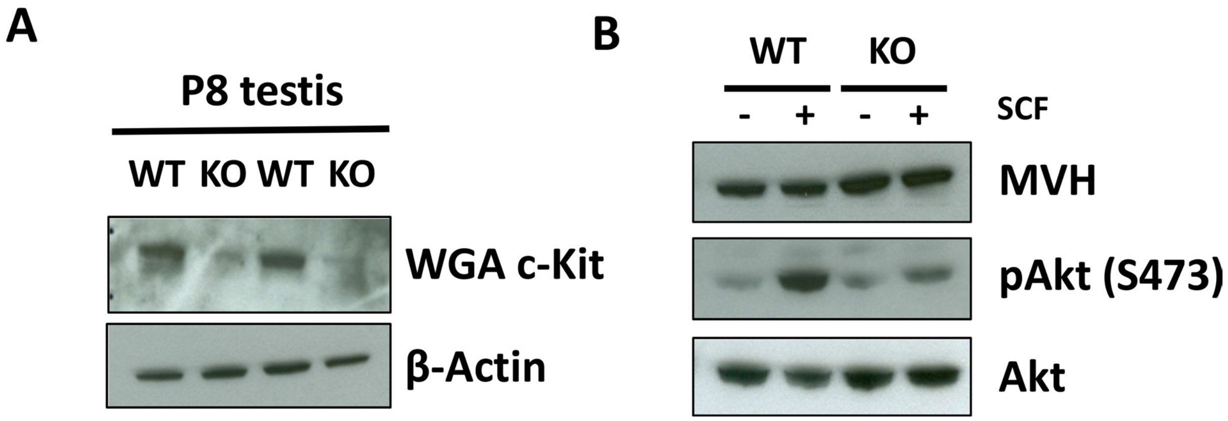

Supplementary Figure S3: Testis MVH levels are not affected by RanBPM deletion. A. Western blot analysis of c-Kit levels in wild type (WT) and RanBPM knockout (KO) Postnatal day 8 (P8) testis after WGA. β-Actin was used as a control in 10% of the input used for the WGA analysis. **B.** MVH and activation of phospho-AKT (pAkt) level analysis in WT and KO dissociated testicular cells treated with 100 ng/mL SCF for 15 minutes. Total Akt was used as loading control.

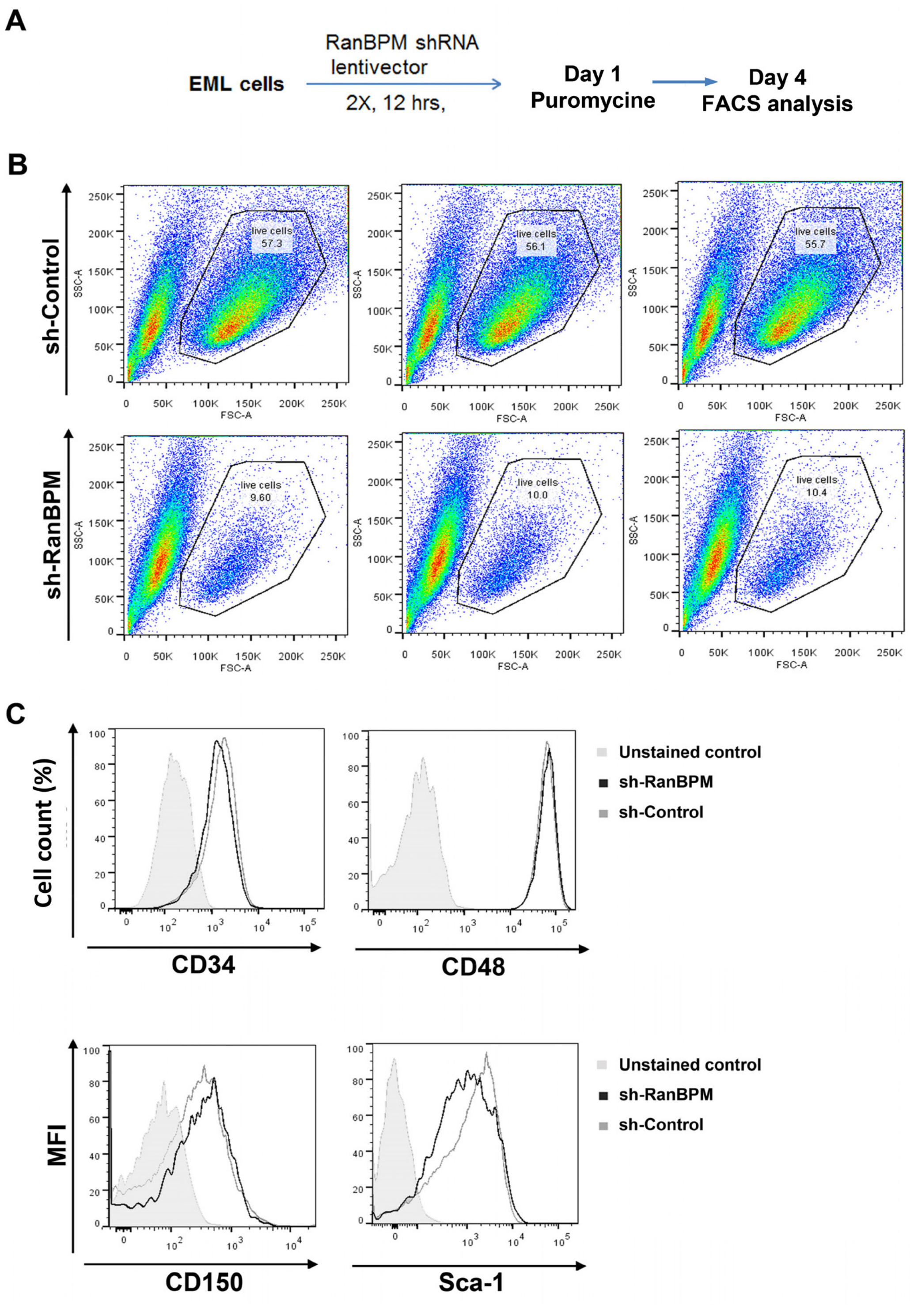

Supplementary Figure S4: RanBPM silencing in EML cells does not affect expression of other stem cell markers. A. Experimental scheme for knockdown of RanBPM in EML cells used in FACS analysis. **B.** Forward and side scatter of control and RanBPM shRNA lentivirus transduced EML cells 4 days after viral transduction. **C.** Mean fluorescence intensity (MFI) of CD34, CD48, CD150 and Sca-1 in live-gated EML cells 4 days after lentivirus transduction. Exponentially growing EML cells in RetroNectin® coated plates were transduced with control or RanBPM shRNA lentiviruses. 0.6×10^6 cells EML cells were transduced two times at 12-hour intervals. Twenty four hours after the last transduction 0.1×10^6/ml cells were seeded in 24 well plates in the presence of 100ng/ml of mSCF and 0.2 µM of puromycin to eliminate non-infected cells. Three days later EML cells were incubated with antibodies that recognize c-Kit, Sca-1, CD34, Flk-2, PE-Cy7-conjugated CD150 and CD48 to determine the levels of cell surface expression by flow cytometry.

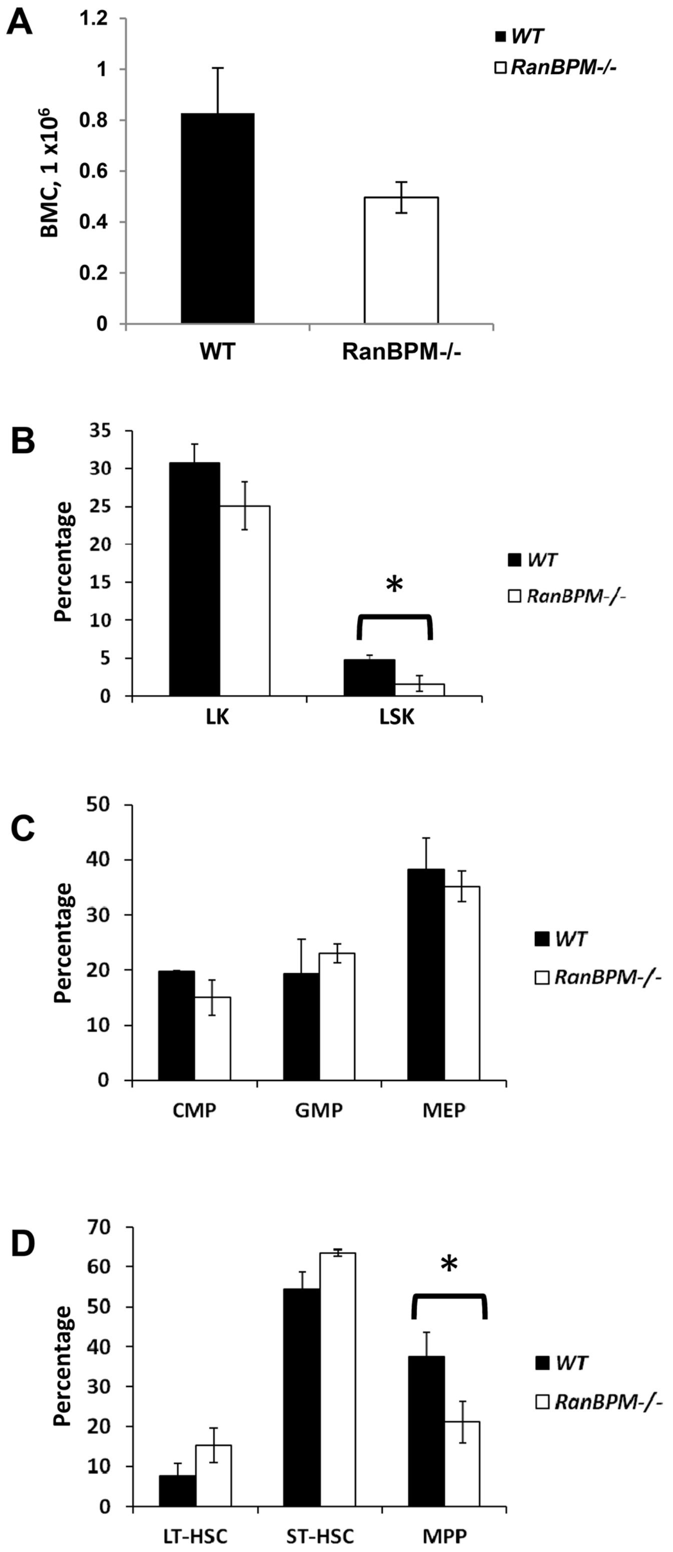

Supplementary Figure S5: RanBPM deficiency does not prevent the development of Lineage-negative cells but causes a reduction in c-kit+ progenitor cells. Bone marrow cellularity **A.** and quantification of flow cytometric analyses of Lineage-negative cells **B.**, LK cells **C.** and LSK cells **D.** from the bone marrow of WT and *RanBPM*$^{-/-}$ mice as in Figure 4B-G. Percentages of gated cell populations within the parent populations are indicated. Graphs show percentages ± SEM. $n = 3$ mice per genotype. * p<0.05.

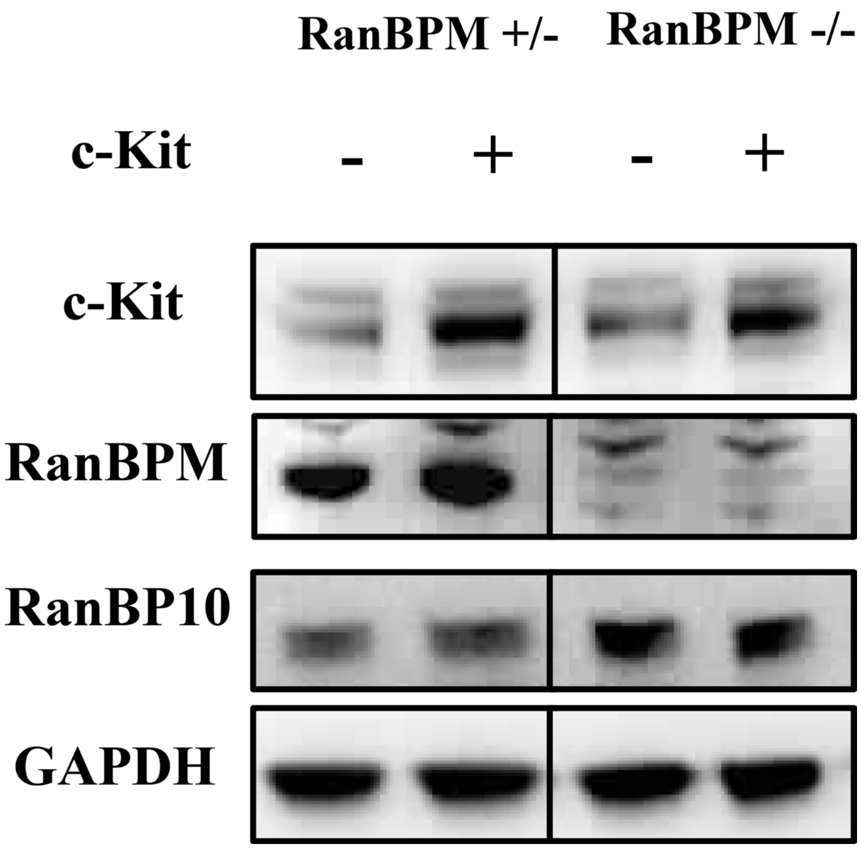

Supplementary Figure S6: RanBP10 is upregulated in ES cells lacking RanBPM. Heterozygous (+/-) and RanBPM deficient (-/-) embryonic (ES) stem cells [11] were transfected with a vector expressing c-Kit and analyzed by Western blot for expression of c-Kit, RanBPM, RanBP10 and GAPDH. Note the normal expression of c-Kit in RanBPM-deficient ES cells with RanBP10 upregulation.
